# On the Use of Electricity in Obstetrics

**Published:** 1870-06

**Authors:** 


### On the Use of Electricity in Obstetrics.

Dr. de St. Germain, surgeon to the Lying-in-Hospital, has recently been conducting a series of experiments with electricity, as an obstetric agent, and he thus sums up the results in a communication brought forward at the Imperial Society of Surgery:—

1. We have not been able in any one case to produce uterine contractions, when they had not already appeared of their own accord.

2. Whenever labor had commenced, and the contractions were succeeding each other at intervals of fifteen or twenty minutes, the application of electricity to the lateral walls of the abdomen produced a considerable amelioration of the uterine contractions, after the lapse of only ten minutes.

2. We have also stated that each contraction excited by electricity was much longer and more painful than the others.

4. The dilation of the cervix seemed to press constantly with rapidity under the influence of galvanic excitement.

5. In all the cases the expulsion of the placenta immediately followed that of the child.

6. In two instances only we observed a slightly bluish hue on the skin of the child; but this might have been attributed to cyanosis, brought on by circular constriction.—*Lancet.*

